# Tubridge flow diverter alone vs. Tubridge flow diverter and coils for the treatment of intracranial aneurysms: A propensity score matching analysis

**DOI:** 10.3389/fneur.2022.974354

**Published:** 2022-12-07

**Authors:** Min Shi, Yu Feng, Cheng-Da Zhang, Qing-Wen Tang, Ze-Jin Li, Wen-Yuan Zhao, Ting-Bao Zhang

**Affiliations:** Department of Neurosurgery, Zhongnan Hospital of Wuhan University, Wuhan, China

**Keywords:** intracranial aneurysms, tubridge flow diverter, coiling, endovascular treatment, aneurysm occlusion rate

## Abstract

**Background:**

The study was designed to assess the clinical performance of a tubridge flow diverter (TFD) in the treatment of intracranial aneurysms and to compare the efficacy and safety between intracranial aneurysms treated with TFD alone and TFD combined with coiling.

**Methods:**

In this retrospective study, patients treated with the TFD alone or TFD combined with coiling between June 2018 to November 2022 were included. The patient demographics, the characteristics of the aneurysm, and the treatment outcomes between the two groups were compared. Propensity score matching was performed to match the variables with a significant difference between groups.

**Results:**

In the current study, data from 93 consecutive patients including 104 aneurysms treated with TFD were analyzed. In total, 43 patients with 49 aneurysms were treated with TFD alone, and 50 patients with 55 aneurysms were treated with TFD combined with coiling. Aneurysms in the TFD combined with the coiling group were larger (12.9 ± 8.6 vs. 8.7 ± 8.8 mm, *P* = 0.016) and more likely to be saccular (92.7% vs. 75.5%, *P* = 0.027) than in the TFD alone group. No significant difference was observed between the two groups in terms of perioperative complication rate. During the follow-up period, the complete occlusion rate in the TFD combined with the coiling group was higher (80.0% vs. 43.8%, *P* = 0.001) than in the TFD alone group. These results were further confirmed using a propensity score matching analysis.

**Conclusion:**

TFD combined with coiling can be a safe and effective alternative option for the treatment of complex aneurysms. Given the potential risks of these therapeutic modalities, thus very careful consideration is required on an individual patient basis.

## Introduction

Since the ISAT trial, endovascular treatment has received much attention for treating patients with intracranial aneurysms, due to the less-invasive procedure, greater efficacy, and safety (1, 2). However, previous endovascular treatment of complex aneurysms still faces numerous challenges such as high rebleeding and recurrence (3, 4). Subsequently, flow-diverter devices (FDDs) have emerged as new embolization devices to treat challenging intracranial aneurysms (5, 6). FDDs are shown to help reconstruct the parent artery and redirect blood flow away from the aneurysm, thus helping treat challenging intracranial aneurysms (5, 6).

Tubridge flow diverter (TFD; MicroPort, Shanghai, China) is a new type of flow-diverter stent, made with nickel-titanium braided microfilament (7). It has greatly advanced the treatment of complex and challenging intracranial aneurysms (8). TFD was developed and advanced based on the theory of flow diverter and successful clinical practice, characterized by self-expandable and high metal coverage (9, 10). However, there were only a few studies that focused on assessing the usage of TFD in intracranial aneurysms. In addition, it is unclear whether the addition of adjunctive coil embolization is required for more efficacy. Some studies have suggested that adjunctive coil embolization can promote thrombosis in the aneurysm sac and enhance the degree of flow diversion, thus improving the occlusion rate of the aneurysm (11). However, some studies suggested that adjunctive coil embolization not only hardly improves the already high occlusion rates but also leads to additional complications due to the overly dense coils inside the aneurysm (12, 13). In the current study, we aimed to assess and compare the clinical performance of TFD alone or TFD combined with coiling in the treatment of intracranial aneurysms.

## Methods and materials

### Ethics approval

This study was approved by the Institutional Review Board (IRB) of Zhongnan Hospital, Wuhan University.

### Study population and clinical data

This is a retrospective single-center study, which was conducted through analysis of data from all consecutive patients admitted to our institution and treated with TFD between June 2018 and November 2021. The study cohort was classified into two groups (TFD alone and TFD combined with coiling groups) based on the presence or absence of coiling, and the two groups were matched using propensity score matching.

Data used for analysis were patient demographic information, including age, sex, comorbidities, clinical presentation, and clinical outcomes at admission; aneurysm characteristics, including aneurysm size, neck size, aneurysm form, location, and aneurysm rupture; perioperative data, including technical success rate, devices used, perioperative complications, and immediate angiographic results; and follow-up data, including complications, clinical outcomes, and angiographic results at final follow-up.

### The interventional procedures and medications

Before the procedure, written informed consent was obtained from all patients. All patients were administered dual antiplatelet management, including 100 mg aspirin once daily and 90 mg ticagrelor twice daily for at least 5 days, and each patient received an antiplatelet resistance test. For patients undergoing urgent or emergency surgery for ruptured aneurysms, tirofiban was administered as an intravenous bolus of 5 μg/kg over a 3-min period as soon as the stent was deployed, followed by a maintenance infusion of 0.06–0.08 μg/kg/min tirofiban for 24 h. Post-tirofiban infusion, a 300 mg loading dose of aspirin or a 300 mg loading dose of clopidogrel was administered. Dual antiplatelet therapy was overlapped with half the tirofiban dose, 2 h before finishing the infusion of tirofiban.

All procedures were performed under general anesthesia or local anesthesia with conscious sedation by one or two experienced interventional neuroradiologists. Based on the jet sign and aneurysm characteristics, the neurosurgeons and the neurointerventionalists decided whether to use coils or not. TFD implantation was performed using systemic devices such as a guiding catheter, an intermediate catheter, and a marksman microcatheter. Before TFD implantation, a coiling microcatheter (echelon-10 or headway-17) was usually superselected into the aneurysm dome upon TFD placement, through which coils are deployed. After the procedure, daily doses of 90 mg of ticagrelor twice daily for 3 months and 100 mg/day of aspirin indefinitely were prescribed.

### Outcome variables

An angiographic imaging was performed immediately after the procedure and during follow-up. The classification of aneurysm embolization was according to the Raymond grading scale (I: complete occlusion; II: residual neck; and III: residual aneurysm) (14).

The clinical outcome assessment was performed at discharge and during follow-up by two experienced neurosurgical staff. The clinical outcome was assessed by the modified Rankin Scale (MRS). The MRS score of 0–2 was regarded as a good outcome, and the MRS score of 3–6 was regarded as a poor outcome.

### Statistical analysis

Data analyses were carried out using SPSS software (SPSS 26.0). Categorical variables were presented as numbers and percentages, while continuous data were expressed using the mean ± standard deviation values. The chi-square and Fisher exact tests were used to compare categorical variables. Student's *t*-test was used to compare continuous data with normally distributed variables, and the Mann–Whitney *U*-test was used to compare continuous data with non-normally distributed variables. The propensity score matching was conducted with a 1:1 matching protocol. The *p*-value of < 0.05 was considered a statistically significant difference.

## Results

### Baseline characteristics

A total of 93 consecutive patients with 104 intracranial aneurysms were treated at our institution between June 2018 and November 2021. Among them, 43 patients with 49 aneurysms were treated with TFD alone, and 50 patients with 55 aneurysms were treated with TFD combined with coiling.

The characteristics of the patients and aneurysms are presented in Table 1. We observed no statistically significant differences in age, sex, smoking, medical comorbidities, presentation, and clinical outcomes between the TFD alone group and the TFD combined with the coiling group at admission. More than half of the patients were female, and hypertension was found to be a common condition in both groups. The majority of patients (66.3%) were symptomatic, and four patients with ruptured aneurysms presented as subarachnoid hemorrhages.

**Table 1:** Patients and aneurysm characteristics before and after propensity score matching.

	**Before propensity score matching**			**After propensity score matching**		
	**TFD alone** **(*n =* 49)**	**TFD + coiling** **(*n =* 55)**	***P***	**TFD alone** **(*n =* 38)**	**TFD + coiling** **(*n =* 38)**	***P***
Age (year)	55.6 ± 11.8	56.1 ± 9.0	0.798	55.7 ± 12.6	54.8 ± 9.4	0.742
Female	57.1% (28/49)	74.5% (41/55)	0.061	60.5% (23/38)	73.7% (28/38)	0.222
Smoking	26.5% (13/49)	16.4% (9/55)	0.205	23.7% (9/38)	15.8% (6/38)	0.387
Hypertension	44.9% (22/49)	43.6% (24/55)	0.897	39.5% (15/38)	36.8 (14/38)	0.813
Diabetes	2.0% (1/49)	5.5% (3/55)	0.620	2.6% (1/38)	5.3% (2/38)	1.000
Stroke	18.4% (9/49)	7.3% (4/55)	0.136	18.4% (7/38)	9.2% (3/38)	0.309
**Presentation**						
Incidental	30.6% (15/49)	29.1% (16/55)	0.866	28.9% (11/38)	28.9% (11/38)	1.000
Symptomatic	65.3% (32/49)	67.3% (37/55)	0.832	65.8% (25/38)	65.8% (25/38)	1.000
Current SAH	4.1% (2/49)	3.6% (2/55)	1.000	5.3% (2/38)	5.3% (2/38)	1.000
Size (mm)	8.7 ± 8.8	12.9 ± 8.6	0.016*	8.2 ± 7.1	9.8 ± 7.3	0.357
Neck size (mm)	5.9 ± 5.5	6.5 ± 3.4	0.507	5.6 ± 5.7	5.3 ± 2.2	0.776
**Aneurysm form**						
Saccular	75.5% (37/49)	92.7% (51/55)	0.027*	89.5% (34/38)	92.1% (35/38)	1.000
Fusiform	6.1% (3/49)	0% (0/55)	0.101	2.6% (1/38)	0% (0/38)	1.000
Dissection	18.4% (9/49)	7.3% (4/55)	0.136	7.9% (3/38)	7.9% (3/38)	1.000
**Location**						
Anterior circulation	83.7% (41/49)	87.3% (48/55)	0.602	94.7% (36/38)	86.8% (33/38)	0.430
Anterior circulation proximal	75.5% (37/49)	85.5% (47/55)	0.199	84.2% (32/38)	86.8% (33/38)	0.744
Anterior circulation distal	8.2% (4/49)	1.8% (1/55)	0.185	10.5% (4/38)	0% (0/38)	0.115
Posterior circulation	16.3% (8/49)	12.7% (7/55)	0.602	5.3% (2/38)	13.2% (5/38)	0.430
VA	16.3% (8/49)	7.3% (4/55)	0.220	5.3% (2/38)	5.3% (2/38)	1.000
BA	0% (0/49)	3.6% (2/55)	0.497	0% (0/38)	5.3% (2/38)	0.493
PICA	0% (0/49)	1.8% (1/55)	1.000	0% (0/38)	2.6% (1/38)	1.000

SAH, subarachnoid hemorrhage; mRS, modified Rankin score; VA, vertebral artery; BA, basilar artery; PICA, posterior inferior cerebellar artery. *Indicates *P* < 0.05.

The mean aneurysm size was 8.7 ± 8.8 mm in the TFD alone group and 12.9 ± 8.6 mm in the TFD combined with the coiling group. For obvious reasons, aneurysm sizes in the TFD combined with the coiling group were significantly larger than in the TFD alone group (*P* = 0.016). We found no statistical difference in the neck size of aneurysms between the two groups (*P* = 0.507). However, there were significantly more saccular aneurysms in the TFD combined with the coiling group compared to the TFD alone group (92.7 vs. 75.5%, *P* = 0.027). No statistically significant difference was observed in the location of aneurysms. Most of the aneurysms were located in the anterior circulation proximal (83.7 vs. 87.3%, *P* = 0.602). Due to these significant differences in the characteristics of the aneurysms between the two groups, we performed a propensity score matching. After propensity score matching using a 1:1 matching protocol, there were 38 patients in each group with comparable baseline characteristics.

### Procedure characteristics

All TFD stent deployments were successful. Three patients in the TFD alone group and four patients in the TFD combined with the coiling group required balloon angioplasty due to incomplete wall apposition. Intraoperative aneurysm rupture occurred in a patient who was treated with TFD combined with coiling. After the procedure, Xper-CT and Digital subtraction angiography (DSA) confirmed the subarachnoid hemorrhage and the dissecting aneurysm rupture of the left vertebral artery V4 segment. Intraprocedural in-stent thrombosis occurred in one patient who was treated with TFD alone and in one patient who was treated with TFD combined with coiling. The DSA showed complete resolution of the thrombus immediately after the tirofiban injection. There were overall four (8.2%) and five (9.1%) ischemic events in the TFD alone and the TFD combined with the coiling group, respectively.

After propensity score matching, three patients (7.9%) in the TFD alone group and two patients (5.3%) in the TFD combined with the coiling group required balloon angioplasty due to incomplete wall apposition. There were two (5.3%) and four (10.5%) ischemic events in the TFD alone and the TFD combined with the coiling group, respectively (*P* = 0.674). Furthermore, the propensity score analysis indicated that there were no significant differences in the overall periprocedural complications between both groups.

### Angiographic evaluation

All patients received immediate postoperative angiograms. In the TFD alone group, all aneurysms presented remarkable stagnation, graded as Raymond III in 100% of patients. Conversely, patients treated with TFD combined with coiling achieved better immediate postoperative angiographic results. A complete occlusion rate was achieved in 3.6% of cases (*P* = 0.497), a near-complete occlusion rate was achieved in 10.9% of cases (*P* = 0.028), and an incomplete occlusion rate of 85.8% was achieved (*P* = 0.006). However, after propensity score matching, no significant differences were observed in Raymond I, Raymond II, and Raymond III grades.

Postprocedural follow-up angiograms were performed at a mean of 8.7 ± 7.2 months in patients of the TFD alone group and a mean of 11.3 ± 8.2 months in patients in the TFD combined with the coiling group. In total, 30.8% (32/104) of aneurysms did not reach the follow-up time or were lost to imaging follow-up. The rate of complete occlusion was higher in the TFD combined with the coiling group than in the TFD alone group (80.0 vs. 43.8%, *P* = 0.001), and the rate of incomplete occlusion was lower in the TFD combined with the coiling group than in the TFD alone group (2.5 vs. 53.1%, *P* < 0.001). No statistically significant difference was observed in the rate of near-complete occlusion between both groups (3.1 vs. 17.5%, *P* = 0.068). The rate of satisfactory aneurysm occlusion (Raymond I/II) was higher in the TFD combined with the coiling group than in the TFD alone group (97.5 vs. 46.9%, *P* < 0.001). Propensity score matching confirmed a higher portion of aneurysms with complete aneurysm occlusion (*P* < 0.001) and with satisfactory aneurysm occlusion (*P* < 0.001) in the TFD combined with the coiling group. Detailed results are shown in Table 2.

**Table 2:** Periprocedural complications and angiography outcomes.

	**Before propensity score matching**			**After propensity score matching**		
	**TFD alone** **(*n =* 49)**	**TFD + coiling** **(*n =* 55)**	***P***	**TFD alone** **(*n =* 38)**	**TFD + coiling** **(*n =* 38)**	***P***
**Periprocedural complication**						
Incomplete wall apposition requiring balloon angioplasty	6.1% (3/49)	7.3% (4/55)	1.000	7.9% (3/38)	5.3% (2/38)	1.000
periprocedural aneurysm rupture	0% (0/49)	1.8% (1/55)	1.000	0% (0/38)	0% (0/38)	1.000
Acute in-stent thrombosis	2.0% (1/49)	1.8% (1/55)	1.000	2.6% (1/38)	0% (0/38)	1.000
Ischemia events	8.2% (4/49)	9.1% (5/55)	1.000	5.3% (2/38)	10.5% (4/38)	0.674
**Immediate angiography**						
Raymond I	0% (0/49)	3.6% (2/55)	0.497	0% (0/38)	2.6% (1/38)	1.000
Raymond II	0% (0/49)	10.9% (6/55)	0.028*	0% (0/38)	10.5% (4/38)	0.115
Raymond III	100% (49/49)	85.5% (47/55)	0.006**	100% (38/38)	86.8% (33/38)	0.054
Follow-up time (months)	8.7 ± 7.2	11.3 ± 8.2	0.181	8.2 ± 6.2	10.8 ± 7.1	0.169
**Follow-up angiography**						
Raymond I	43.8% (14/32)	80.0% (32/40)	0.001**	30.4% (7/23)	81.3% (26/32)	< 0.001**
Raymond II	3.1% (1/32)	17.5% (7/40)	0.068	4.3% (1/23)	15.6% (5/32)	0.383
Raymond III	53.1% (17/32)	2.5% (1/40)	< 0.001**	65.2% (15/23)	3.1% (1/32)	< 0.001**
Satisfactory results	46.9% (15/32)	97.5% (39/40)	< 0.001**	34.8% (8/23)	96.9% (31/32)	< 0.001**
Aneurysm recanalization	0% (0/49)	0% (0/55)	1.000	0% (0/38)	0% (0/38)	1.000
In-stent stenosis at follow-up	9.4% (3/32)	12.5% (5/40)	0.725	5.3% (2/38)	10.5% (4/38)	0.674

*Indicates *P* < 0.05; **Indicates *P* < 0.01.

During follow-up, in-stent stenosis was observed in three patients in the TFD alone group and five patients belonging to the TFD combined with the coiling group. In three patients of the TFD alone group, one patient presented with moderate stenosis at the 3-month follow-up angiogram, which changed to mild stenosis at the 1-year follow-up angiogram, and the other two patients had mild stenosis. In five patients of the TFD combined with the coiling group, two patients presented with moderate stenosis, and three patients had mild stenosis. At the second follow-up, none of the patients who previously had moderate stenosis was documented with in-stent stenosis. Moreover, after propensity score matching, there were no significant differences in the incidence of in-stent stenosis between the TFD alone group and the TFD combined with the coiling group (*P* = 0.674).

**Figure 1:**
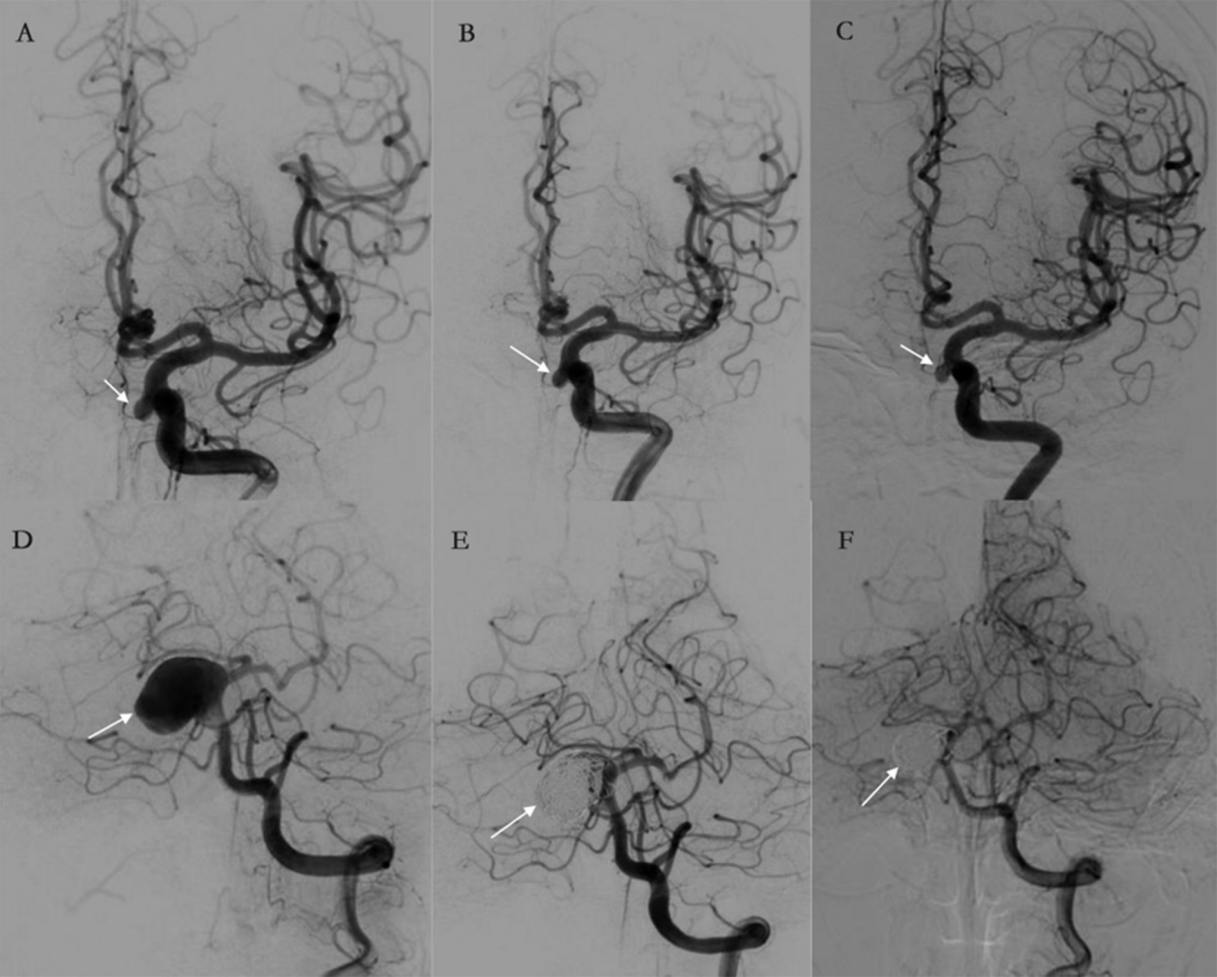
**(A–F)** Illustrations of angiographic results in a case treated by the Tubridge flow diverter (TFD) alone **(A–C)** and a case treated by TFD combined with coiling **(D–F)**. The arrows point to target aneurysms. **(A–C)** An aneurysm treated by TFD alone at pre-treatment, post-treatment, and follow-up. The angiography results show that the aneurysm was incompletely occluded and are classified as Raymond III at 6 months. **(D–F)** An aneurysm treated by TFD combined with coiling at pre-treatment, post-treatment, and follow-up. The angiography results show complete aneurysm occlusion during a 6-month follow-up.

Angiographic imaging in a subject treated with TFD alone and a subject treated with TFD combined with coiling is shown in Figure 1. These results clearly show that the aneurysms treated with TFD combined with coiling could achieve complete occlusion (Raymond I) 6 months post-surgery, while the aneurysms remained Raymond III when treated by TFD alone 6 months post-surgery.

### Clinical outcomes and mortality

At discharge, good functional outcomes (MRS 0–2) were observed in 48 patients (98.0%) in the TFD alone group and 54 patients (98.2%) in the TFD combined with the coiling group. Of note, no patients were lost to clinical follow-up. Good functional outcomes were observed in 47 patients (95.9%) in the TFD alone group and 51 patients (92.7%) in the TFD combined with the coiling group. The total mortality rate was 4.8% (5/104) in the follow-up period, and there was no difference between the groups in mortality (*P* = 1.000) (Table 3).

**Table 3:** Clinical outcomes.

	**Before propensity score matching**			**After propensity score matching**		
	**TFD alone** **(*n =* 49)**	**TFD + coiling** **(*n =* 55)**	***P***	**TFD alone** **(*n =* 38)**	**TFD + coiling** **(*n =* 38)**	***P***
**Clinical outcome at discharge**						
Good clinical outcome	98.0% (48/49)	98.2% (54/55)	1.000	97.4% (37/38)	100% (38/38)	1.000
Poor clinical outcome	2.0% (1/49)	1.8% (1/55)	1.000	2.6% (1/38)	0% (0/38)	1.000
Mortality at discharge	0% (0/49)	0% (0/55)	1.000	0% (0/43)	0% (0/50)	1.000
**Clinical outcome at follow-up**						
Good clinical outcome	95.9% (47/49)	92.7% (51/55)	0.681	94.7% (36/38)	97.4% (37/38)	1.000
Poor clinical outcome	4.1% (2/49)	7.3% (4/55)	0.681	5.3% (2/38)	2.6% (1/38)	1.000
Mortality at follow-up	4.1% (2/49)	5.5% (3/55)	1.000	5.3% (2/38)	0% (0/38)	0.493

After propensity score matching, there were no significant differences in clinical outcomes between both groups (Table 3).

## Discussion

In recent years, TFD has been gradually introduced into the field of neurointerventional surgery (15, 16). However, there is a lack of sufficient preclinical studies and clinical trials that assessed the efficacy and safety of TFD in the treatment of intracranial aneurysms. In addition, there are no studies to explore whether the use of adjunctive coil embolization with TFD is a more effective and safe treatment modality. In the current study, we conducted a retrospective analysis comparing the clinical performance between TFD alone and TFD combined with coiling. Our results found that TFD combined with coiling can significantly improve the occlusion rate, without increasing the additional complication, which further confirmed the efficacy and safety of TFD alone or combined with coiling for the treatment of intracranial aneurysms.

A tubridge flow diverter achieves occlusion of aneurysms by reconstructing the parent artery. The change in hemodynamic parameters at the parent artery and the aneurysm sac is considered to be the main mechanism (17). TFD can redirect the blood flow and exclude aneurysms from blood circulation, which can lead to subsequent intra-aneurysmal thrombosis (18, 19). With the support offered by aneurysmal thrombosis and blood flow reduction within the aneurysm, endothelialization occurs followed by the placement of flow diverters. The new endothelium forms over the device surface and across the aneurysm neck. This finally leads to the reconstruction of the parent artery vessel and the healing of the aneurysm. According to an early study of TFD by Zhou et.al, the overall complete occlusion rate was 72% during the mean 9.9-month follow-up periods (20). In addition, Liu et al. conducted a multicenter randomized controlled trial. The authors found that TFD treatment achieved a higher rate of large and giant aneurysm obliteration compared to enterprise stent-assisted coiling (15).

To date, there is no consensus on the use of adjunctive coil embolization with TFD. Some researchers had advocated that adjunctive coil embolization can be used as a method for improving occlusion rates and minimizing the potential for catastrophic aneurysm rupture while using flow-diverting stents. However, others have argued that the addition of coil embolization to the procedure yields no significant advantage in terms of treatment efficacy (21). In recent years, many researchers have been involved in studies to confirm the efficacy of FDDs as a stand-alone treatment modality for intracranial aneurysms. However, considering that the coils can accelerate thrombosis of the aneurysmal sac, coils in conjunction with FDD could achieve faster and more satisfactory occlusion results. Some studies have started to explore the effectiveness and safety of coils in conjunction with FDD and compared the effectiveness and safety of FDD alone and coils in combination with FDD. Zhang et al. compared 99 aneurysms with a pipeline embolization device (PED) and 41 aneurysms with PED combined with coiling. In these cases, the adjunctive coil embolization group obtained higher satisfactory angiography results (92.7 vs. 78.8%, *P* = 0.047) without increasing any periprocedural complications (22).

In our study, we observed that the aneurysms in the TFD combined with the coiling group are larger and wider. In addition, adjunctive coil embolization is observed more easily in a saccular aneurysm. For larger and wider aneurysms, neurointerventionalists usually tend to combine TFD and coil embolization. The larger aneurysms have a higher risk of spontaneous rapture. TFD embolization requires a long time to achieve complete occlusion, which can increase the risk of rupture of large or giant aneurysms (23). In such instances, neurointerventionalists add coil embolization to increase stasis. The concurrent use of coils can accelerate thrombus formation in the aneurysmal sac, which can help the aneurysm heal and prevent it from further rupture (24). However, adjunctive coil embolization also increases the complexity of the operation and the overall complication. In a study by Siddiqui et al. (23) a patient with a giant aneurysm was treated with PED combined with dense coil embolization and had an acute thrombosis of the PED immediately after the procedure due to the dense coil mass (23). In contrast, we did not observe any such obvious technique-related complication regarding coiling, mainly due to low coil-packing densities and stringent antiplatelet management.

Similar to previous studies, our present results demonstrated that coiling in conjunction with TFD could obtain a higher occlusion rate for intracranial aneurysms, especially large or giant aneurysms. Our results further confirmed that coiling and TFD are complementary procedures rather than competitive. Therefore, TFD combined with coiling is considered a more effective way in suitable cases due to speeding thrombosis. The discrepancy between these two modalities is not difficult to explain. First, adjunctive coil embolization with the flow diversion technique can promote thrombus formation in the aneurysmal sac, thereby achieving a higher complete occlusion rate and preventing aneurysm rupture (25). Second, the change in flow hemodynamics caused by the deployment of coils can contribute to neointimal hyperplasia formation across the device construct and aneurysm orifice (22). Notably, the deployment of coils affects stent visibility and increases the complexity of the procedure. Moreover, overly dense coils may increase the mass effect and cause additional complications.

We acknowledge that there were some limitations to this study. First, this is a retrospective study, and the number of included participants was small, which may lead to selection bias and could limit the power of our conclusions. Second, adequate follow-up time is required to assess the occlusion rate accurately. However, some patients included in the current study have a shorter follow-up time, which may not accurately reflect the obliteration rates. Third, this is not a multicenter study, and hence, it only represents the experience of a single center. Finally, we did not randomize the patients as to whether or not to use adjunctive coils, as this was entirely the attending surgeon's decision. Therefore, we need to conduct more clinical trials to validate the findings of this study.

## Conclusion

A Tubridge flow diverter combined with coiling can be a safe and effective alternative option for the treatment of a complex aneurysm. In this study, we found that patients who were treated with TFD combined with coiling showed a higher aneurysm occlusion rate without increasing the periprocedural complications. However, given the potential risks of these therapeutic modalities, careful consideration is required on a case-by-case basis. In the future, more clinical and basic experimentations are required to determine the role of coils in TFD-treated cases to guide clinical decisions.

## Data availability statement

The original contributions presented in the study are included in the article/supplementary material, further inquiries can be directed to the corresponding authors.

## Ethics statement

The studies involving human participants were reviewed and approved by the Medical Ethics Committee of Zhongnan Hospital of Wuhan University. The patients/participants provided their written informed consent to participate in this study. Written informed consent was obtained from the individual(s) for the publication of any potentially identifiable images or data included in this article.

## Author contributions

MS took responsibility for the integrity of the data and the accuracy of the data analysis. MS and YF contributed significantly to data analysis, data acquisition, and manuscript preparation. MS, T-BZ, YF, C-DZ, Q-WT, and Z-JL made critical revisions to the manuscript for important intellectual content. W-YZ and T-BZ guided the research and contributed to supervision. All authors contributed to the conception, design, analysis, interpretation of the data of the study, and approved the submitted version.
